# Melatonin inhibits proliferation, migration, and invasion by inducing ROS-mediated apoptosis via suppression of the PI3K/Akt/mTOR signaling pathway in gallbladder cancer cells

**DOI:** 10.18632/aging.203561

**Published:** 2021-09-27

**Authors:** Kunlun Chen, Pengfei Zhu, Wenhui Chen, Kai Luo, Xiao-Jing Shi, Wenlong Zhai

**Affiliations:** 1 Department of Hepatobiliary and Pancreatic Surgery, The First Affiliated Hospital of Zhengzhou University, Zhengzhou, Henan Province 450052, PR China; 2 Laboratory Animal Center, State Key Laboratory of Esophageal Cancer Prevention and Treatment, Academy of Medical Science, Zhengzhou University, Zhengzhou, Henan Province 450052, PR China

**Keywords:** melatonin, apoptosis, PI3K/Akt, ROS, gallbladder cancer

## Abstract

Background: Melatonin is an indolic compound mainly secreted by the pineal gland and plays a vital role in the regulation of circadian rhythms and cancer therapy. However, the effects of melatonin in gallbladder cancer (GBC) and the related mechanism remain unknown.

Methods: In this study, the antitumor activity of melatonin on gallbladder cancer was explored both *in vitro* and *in vivo*. After treatment with different concentrations of melatonin, the cell viability, migration, and invasion of gallbladder cancer cells (NOZ and GBC-SD cells) were evaluated by CCK-8 assay, wound healing, and Transwell assay.

Results: The results showed that melatonin inhibited growth, migration, and invasion of gallbladder cancer cells. Subsequently, the assays suggested that melatonin significantly induced apoptosis in gallbladder cancer cells and altered the expression of the apoptotic proteins, including Bax, Bcl-2, cytochrome C, cleaved caspase-3, and PARP. Besides, the intracellular reactive oxygen species (ROS) was found to be upregulated after melatonin treatment in gallbladder cancer cells. Melatonin was found to suppress the PI3K/Akt/mTOR signaling pathway in a time-dependent manner by inhibiting the phosphorylation of PI3K, Akt, and mTOR. Treatment with N-acetyl-L-cysteine (NAC) or 740 Y-P remarkably attenuated the antitumor effects of melatonin in NOZ and GBC-SD cells. Finally, melatonin suppressed the growth of GBC-SD cells in an athymic nude mice xenograft model *in vivo*.

Conclusions: Our study revealed that melatonin could induce apoptosis by suppressing the PI3K/Akt/mTOR signaling pathway. Therefore, melatonin might serve as a potential therapeutic drug in the future treatment of gallbladder cancer.

## INTRODUCTION

Gallbladder cancer is reported to be the most aggressive and common pathological type of biliary tract cancer word widely. And surgical resection is reported to be the only potentially curative approach [1, 2]. Unfortunately, majority of gallbladder cancer patients are diagnosed when they are at advanced stages, since patients present with metastasis and other symptoms at a late stage [3, 4]. Previous studies report that the 5-year survival rate for GBC is 13%, and the median survival time is below 1 year [5]. Therefore, novel drugs and therapeutic targets for inoperable patients with GBC are urgently needed.

Melatonin has been identified as a crucial amine hormone that is secreted by the pineal gland and gastrointestinal tract. Melatonin has been reported to regulate the circadian rhythm and immune functions [6, 7]. The synthesis and secretion of melatonin are controlled by the light/night clock, meaning that light suppresses melatonin synthesis while darkness stimulates its production. After hydroxylation and decarboxylation, tryptophan synthesizes serotonin (5-hydroxytryptamine) and this process is regulated by tryptophan hydroxylase and decarboxylase [8]. Serotonin is then acetylated, methylated, and converted to melatonin in the pineal gland [9]. Recently, accumulating evidence has revealed that melatonin suppresses tumorigenesis, metastasis, and drug resistance in multiple cancers [10–12]. Melatonin restrains the nuclear translocation of NF-κB to prevent excessive proliferation in lung and liver cancers [13, 14]. Melatonin partly induces apoptosis in pancreatic cancer by upregulating Bax expression [15]. By reducing the expression of iNOS and COX-2, melatonin restricts inflammatory damaging effects, thus inhibiting tumor progression in breast cancer [16]. Melatonin disrupts the tumor blood vessel formation in renal adenocarcinoma by decreasing VEGF [17]. The relationship between melatonin and gallbladder cancer has however not been clearly established.

We evaluated the inhibitory effects of melatonin on gallbladder cancer cell proliferation. Melatonin induced cell apoptosis by suppressing the PI3K/Akt/mTOR signaling pathway. Therefore, melatonin might be an effective treatment for gallbladder cancer.

## MATERIALS AND METHODS

### Reagents and antibodies

Melatonin (HY-B0075) and 740 Y-P (HY-P0175) were bought from MedChemExpress (MCE, China). A 1mol/L stock solution was attained by dissolving melatonin in dimethyl sulfoxide (DMSO), which was then kept at −20°C in the dark. The DMSO and N-acetyl-L-cysteine (NAC) were provided by the Beyotime (Beyotime Institute of Biotechnology, Shanghai, China). The Cell Counting Kit-8 (CCK-8) was bought from US EVERBRIGHT INC. The primary antibodies used in this study included Bax (ab32503, Abcam, Cambridge, MA, USA), Bcl-2 (ab32124, Abcam, Cambridge, MA, USA), Cytochrome C (ab76237, Abcam, Cambridge, MA, USA), Cleaved Caspase-3 (9662, Cell Signaling Technology Inc, CST, MA, USA), PI3K (AB3263, Technology, Shanghai, China), phospho-PI3K (CY6427), phospho-Akt (AY0421), and Phospho-mTOR (CY5996). The HRP-linked goat anti-mouse and anti-rabbit secondary antibodies were also bought from CST.

### Cell lines and culture

Gallbladder cancer cell lines (NOZ and GBC-SD) were acquired from the Cell Bank of the Chinese Academy of Sciences (Shanghai, China).

They were incubated in Dulbecco’s Modified Eagle Medium and RPMI-1640 medium (Solarbio Life Science, Beijing, China) with 10% fetal bovine serum (FBS; HyClone, Utah, USA), 100 mg/L streptomycin and 100 U/mL penicillin. Incubation at 37°C was done in a 5% CO_2_ atmosphere. Logarithmic growth phase cells were obtained and used in the experiment.

### Cell viability assay

The anti-proliferative effects of melatonin were detected by CCK-8 assay in gallbladder cancer cells. Briefly, the NOZ and GBC-SD cells were seeded in 96-well plates (5 × 10^3^/well) followed by treatment with 200 μL cell culture medium containing varying concentrations of melatonin (0, 0.5, 0.75, 1, 1.5, 2, 2.5, and 3 mM) for 24 h. Then the GBC-SD and NOZ cells were subjected to 1 mM melatonin treatment for different time (0, 12, 24, and 48 h). Then, the pre-treatment with 2 mM NAC for 1 h was conducted before treatment of the GBC-SD and NOZ cells with 1 mM melatonin. Co-treatment with melatonin (1 mM) and 740 Y-P (30 uM) for 48 h were also conducted. Finally, CCK-8 (10 μL) was added to each of the wells and the cells were cultured for 2 h. Optical density (OD) at 450 nm was measured by a Varioskan LUX Multimode Microplate Reader (Thermo Fisher Scientific, USA).

### Colony formation assay

The NOZ and GBC-SD cells were cultured in 6-well plates (1 × 10^3^ cells/well) with or without treatment of 1 mM melatonin. After 2 weeks incubation, PBS was used to wash the 6-well plates after which they were methanol stained. Then, staining of colonies was done using crystal violet solution and counted.

### Wound healing assay

The gallbladder cancer cells were harvested and cultured in 6-well plates. At an 85% cell density, a wound was scratched using a 200 μL plastic tip along the scratch line as described [18]. GBC-SD and NOZ cells were cultured in a medium with 2% FBS with melatonin (1 mM). PBS was used to wash the cells, twice, to remove the cell debris. Finally, photos were taken at 0 and 48 h to calculate wound closure percentage.

### Cell migration and invasion assay

The tumor cell migration analysis was detected by Transwell filters (Corning, NY, USA) while invasion analysis was done using the Matrigel invasion chamber (BD Biosciences, NJ, USA) as previously described [19]. Pretreatment of GBC-SD and NOZ cells was done using melatonin (1 mM) for 24 h. Cells were digested, resuspended, and seeded in the upper chamber with serum-free media (200 μL). Then, the lower chamber was supplemented with 500 μL of the medium (20% FBS) followed by 24 h of incubation. Finally, cells that transferred to the lower chamber were fixed and stained using 1% crystal violet. Images were obtained from five random fields to count the cells.

### Cell apoptosis assay

Cell apoptosis rate was assessed using YF^®^488-Annexin V/PI double staining Apoptosis Kit (US EVERBRIGHT INC., San Ramon, USA). Briefly, 2 ml GBC-SD and NOZ cells suspension were cultured in six-well plates (1 × 10^5^ cells/well) and incubated in the presence of melatonin (1 mM) for 48 h. The harvested cells were washed twice using PBS and stained using binding buffer (500 μL) containing 5 μL PI and 5 μL YF^®^488-Annexin V for 15 min in the dark [20]. Sample assessments were finally done using a FACSCanto^™^ Flow Cytometer (BD Biosciences, San Jose, USA) and apoptotic cell percentage computed.

### Measurement of cellular reactive oxygen species (ROS)

Intracellular ROS level was assessed with the Reactive Oxygen Species Assay Kit (Beyotime Institute of Biotechnology, China). Briefly, the tumor cells were cultured in 6-well plates followed by melatonin (1 mM) treatment for 48 h. Then, cells were incubated with the serum-free medium supplemented with DCFH-DA (10 μM). After 30 min, cells were washed twice using PBS and imaged by fluorescent microscopy (BX63, Olympus Corporation, Japan) at 488 nm excitation wavelength.

### Western blot analysis

Treated gallbladder cancer cells were obtained and then lysed with RIPA Buffer (Beyotime Institute of Biotechnology, China) supplemented with 1 mM Phenylmethanesulfonylfluoride (PMSF). After denaturation at 100°C for 10 min, concentrations of proteins were assessed with the BCA Protein Assay Kit (#7780, Cell Signaling Technology Inc, CST, MA, USA) as described [21]. The equal total protein (30 μg) amounts were loaded to SDS-PAGE gel (10%) and blotted onto a nitrocellulose (NC) membrane (Millipore, Merck KGaA, Darmstadt, Germany). After blocking with skim milk (5%), the NC membrane was immunoblotted at 4°C in the presence of primary antibodies overnight. Then, the membrane was washed, incubated for 2 h in the presence of a HRP-conjugated secondary antibody. Finally, the protein bands were visualized with the Image Lab system (Bio-Rad Laboratories, Inc.,). Expression levels of GAPDH or beta-actin were used as controls.

### Xenograft studies

The male BALB/c-nu mice (6 weeks old) were procured from Hunan Slack Scene of Laboratory Animal Co., Ltd (Hunan, China) and raised in a specific pathogen-free (SPF) environment. This study was permitted by the Ethical Committee of Experimental Animals of Zhengzhou University. 5 × 10^6^ GBC-SD cells in PBS (200 μL) were subcutaneously administered into the right-back of every mouse [22]. Then, mice were randomized into two groups (six mice in each group) after which treatment was initiated at a tumor volume of 40 mm^3^. Saline and 5 mg/kg melatonin were injected intraperitoneally into GBC-SD-bearing mice daily. Mice weights as well as tumor volumes were assessed every 3 days. At a tumor volume of about 900 mm^3^, mice were sacrificed and tumor tissues weighed.

### Statistical analysis

Analyses were done using the SPSS software (version 23.0, Chicago, IL, USA). Comparison of means between groups was done by the Unpaired Student’s *t*-test. Three biological replicates were performed and significance was determined at *p* ≤ 0.05, unless otherwise specified.

### Availability of data and materials

The data generated and analyzed during the current study are available from the corresponding author on a reasonable request.

## RESULTS

### Melatonin reduces GBC-SD and NOZ cells cell viability

**Figure 1:**
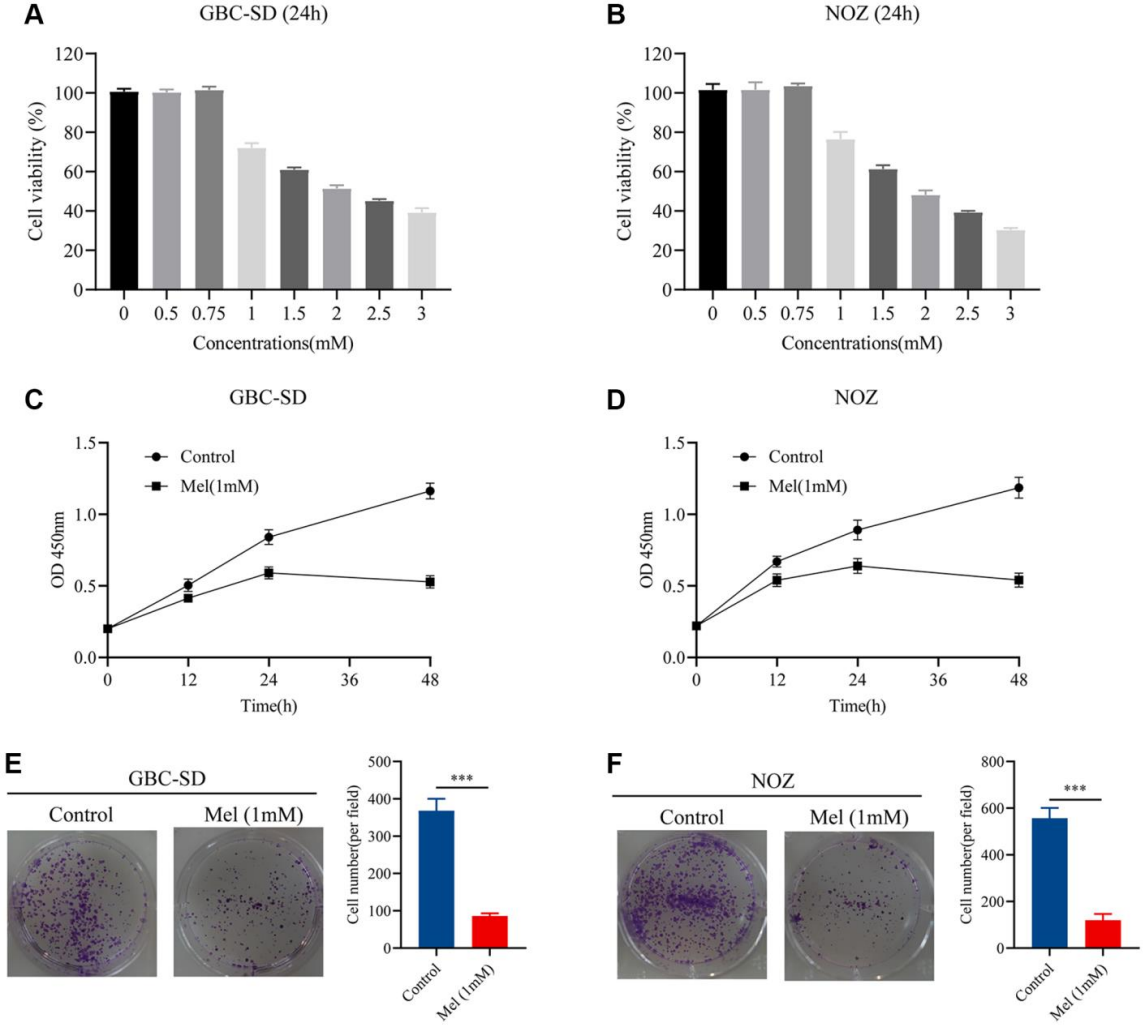
**Melatonin inhibits proliferation in GBC-SD and NOZ cells.** (**A**) Cell viability of GBC-SD cells after treatment with different melatonin concentrations (0, 0.5, 0.75, 1, 1.5, 2, 2.5, and 3 mM) for 24 hours. (**B**) Cell viability of NOZ cells after treatment with different melatonin concentrations (0, 0.5, 0.75, 1, 1.5, 2, 2.5, and 3 mM) for 24 hours. (**C**) Cell viability of GBC-SD cells after treatment with 1 mM melatonin at different times (0, 12, 24, 48 h) by CCK-8 assay. (**D**) Cell viability of NOZ cells after treatment with 1 mM melatonin at different times (0, 12, 24, 48 h) by CCK-8 assay. (**E**) Colony formation assay of GBC-SD cells with or without 1 mM melatonin treatment for 14 days. (**F**) Colony formation assay of NOZ cells with or without 1 mM melatonin treatment for 14 days. Three biological replicates were performed. Data are presented as mean ± SD. Mel, melatonin; ^***^P < 0.001.

Gallbladder cancer cells were treated with varying melatonin concentrations (0, 0.5, 0.75, 1, 1.5, 2, 2.5, and 3 mM) for 48 h. To identify the anti-proliferative effect of melatonin on NOZ and GBC-SD cells, we detected the cell viability using the CCK-8 assay (Figure 1A, 1B). Tumor cells were then treated with melatonin (1 mM) at different times (0, 12, 24, and 48 h). The results showed that 1 mM melatonin dose-dependently markedly inhibited the cell viability of gallbladder cancer cells (Figure 1C, 1D). Moreover, the colony formation experiment revealed that treatment with melatonin (1 mM) suppressed cell clonogenicity in NOZ and GBC-SD cells (Figure 1E, 1F). Therefore, 1 mM was selected as the appropriate concentration for subsequent experiments.

### Melatonin inhibits gallbladder cancer cells motility and invasion

**Figure 2:**
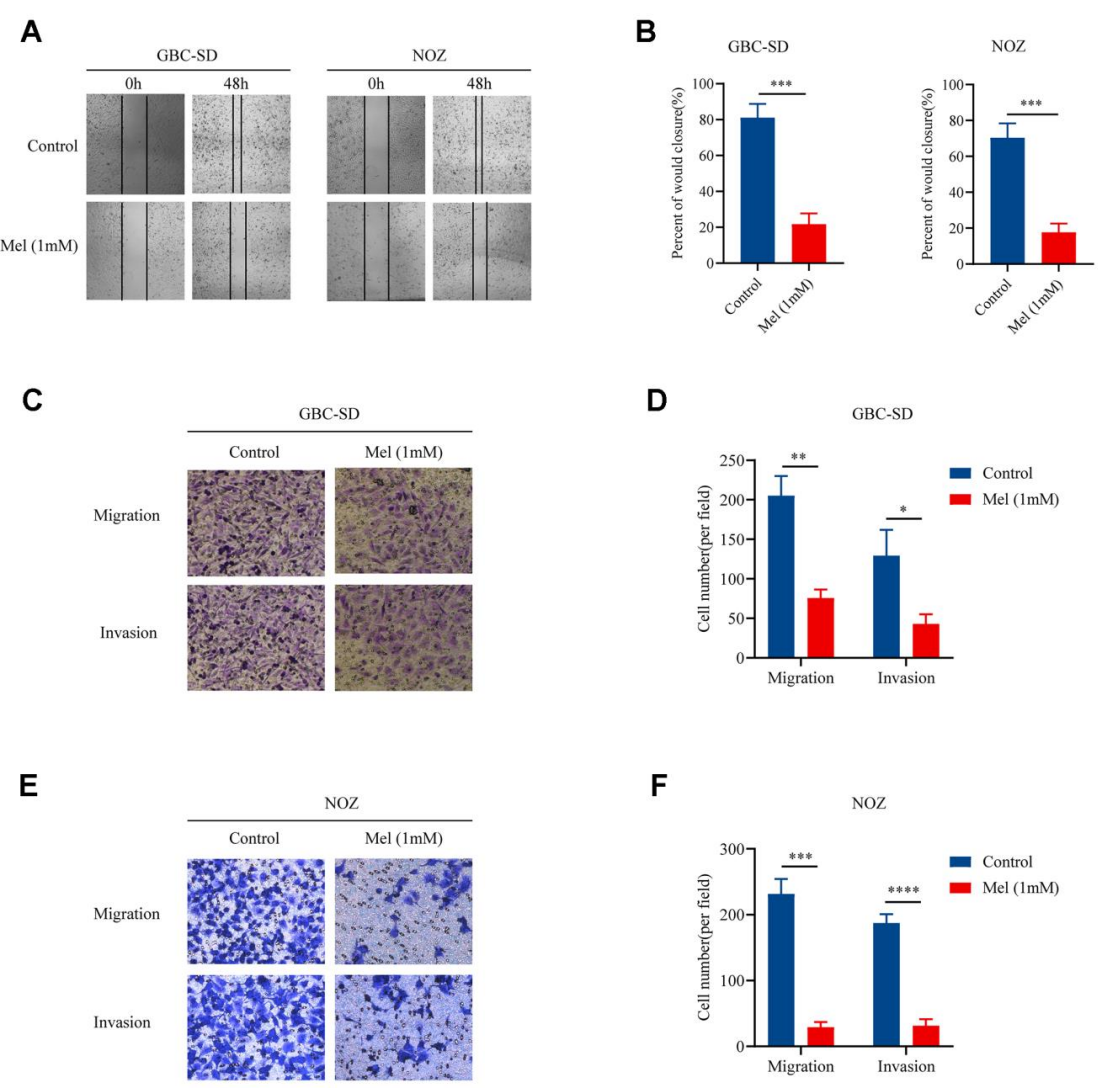
**Melatonin suppresses the migration and invasion of gallbladder cancer cells.** (**A**) The wound-healing assay in GBC-SD and NOZ cells treated with or without 1 mM melatonin for 48 h. (**B**) The percentage of wound closure in GBC-SD and NOZ cells. (**C**) The migration and invasion assay in GBC-SD cells treated with or without 1 mM melatonin. (**D**) Transwell assays assessed GBC-SD cell number per filed. (**E**) The migration and invasion assay in NOZ cells treated with or without 1 mM melatonin. (**F**) Transwell assays assessed NOZ cell number per filed. Three biological replicates were performed. Data are presented as mean ± SD. Mel, melatonin; ^***^P < 0.001; ^**^P < 0.01; ^*^P < 0.05.

Since cellular motility and invasiveness are key steps in cancer metastasis, we examined the motility as well as invasion of gallbladder cancer cells treated with melatonin (1 mM). Treatment with melatonin (1 mM) restrained the movement of NOZ and GBC-SD cells (Figure 2A). Mean percentage of wound closure was approximately 21.7% and 17.7%, respectively (Figure 2B). In the Transwell assay, both tumor cell migration as well as invasion abilities were restricted. The results in Figure 2C and 2D suggest that fewer GBC-SD cells could traverse the membrane when treated with melatonin (1 mM). And melatonin (1 mM) significantly decreased the migration as well as invasive abilities of NOZ cells (Figure 2E, 2F). Taken together, the data showed that melatonin successfully suppressed gallbladder cancer cell motility as well as invasion.

### Melatonin promotes ROS production and apoptosis induction in gallbladder cancer cells

**Figure 3:**
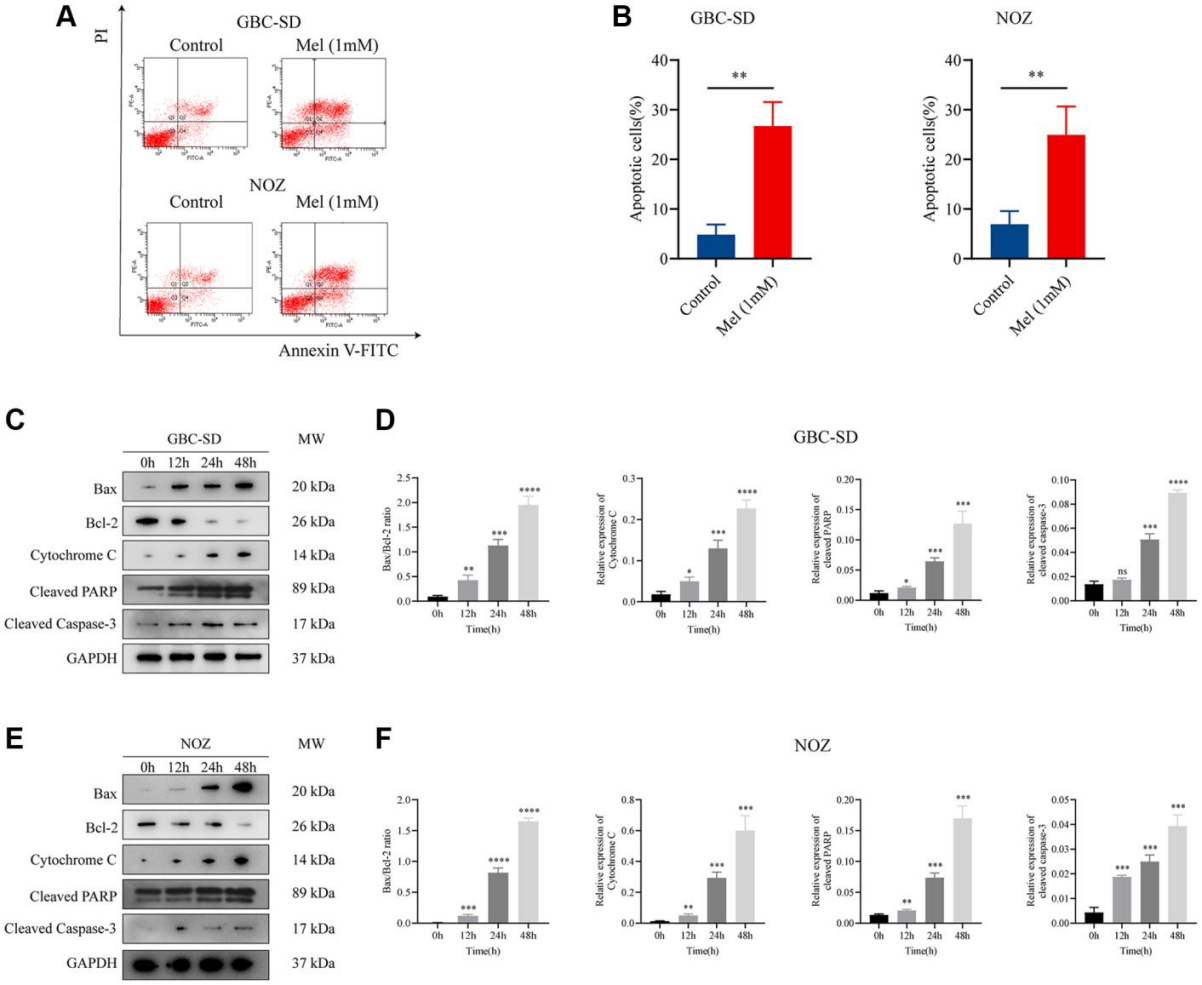
**Melatonin induces apoptosis in gallbladder cancer cells.** (**A**) Apoptosis of GBC-SD and NOZ cells treated with 1 mM melatonin was analyzed by flow cytometry. (**B**) The percentage of apoptotic cells of GBC-SD and NOZ cells was quantified. (**C**) Expression of Bax, Bcl-2, cytochrome C, cleaved PRRP, and cleaved caspase-3 was investigated by Western blot after GBC-SD cells were treated with 1 mM melatonin. (**D**) The relative expression of the apoptotic markers was quantified in GBC-SD cells. (**E**) Expression of Bax, Bcl-2, cytochrome C, cleaved PRRP and cleaved caspase-3 was investigated by Western blot after NOZ cells were treated with 1 mM melatonin. (**F**) The relative expression of the apoptotic markers was quantified in NOZ cells. Three biological replicates were performed. Data are presented as mean ± SD. Mel, melatonin; ^***^P < 0.001; ^**^P < 0.01; ^*^P < 0.05; ns, no significance.

**Figure 4:**
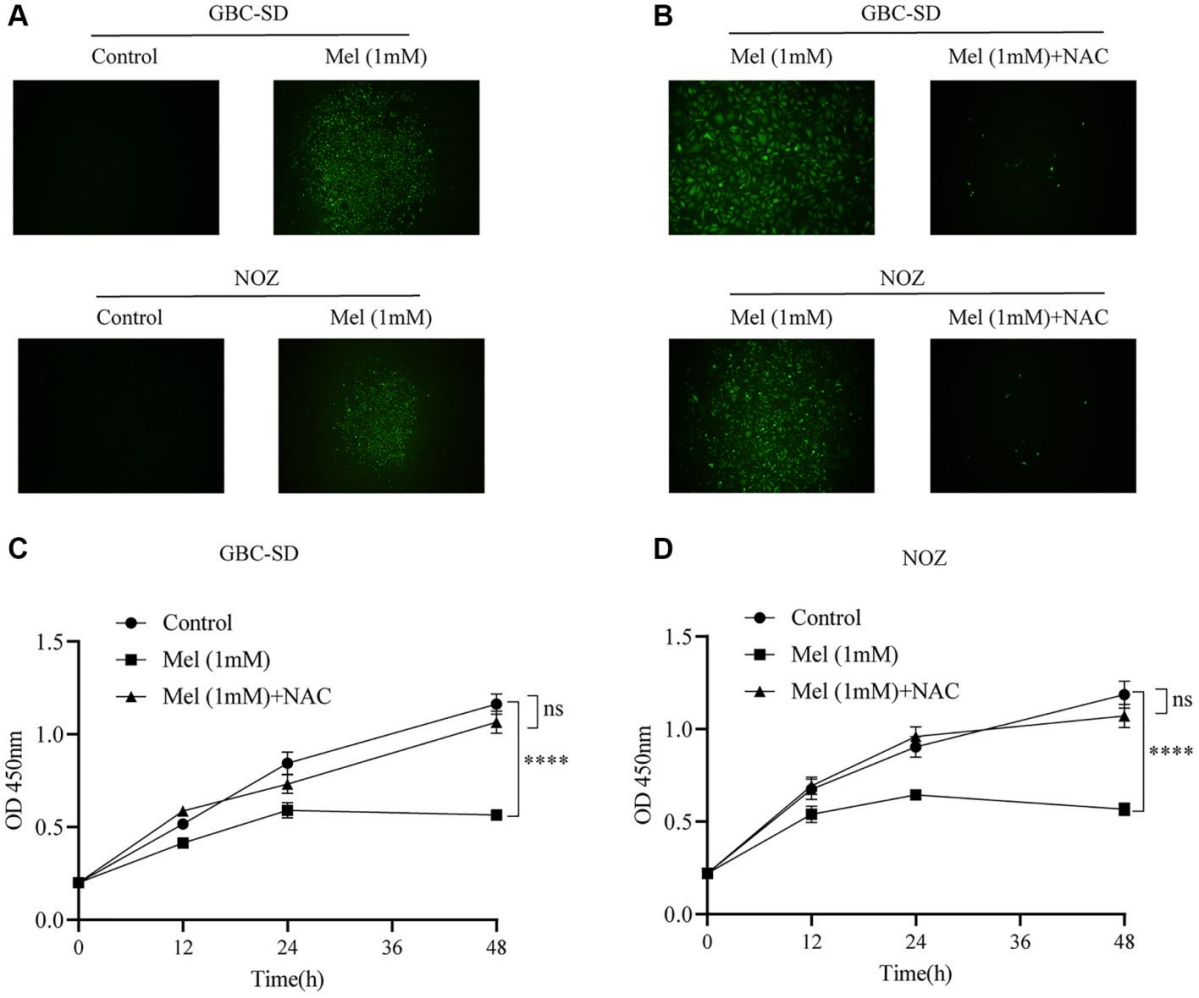
**The level of ROS increases after melatonin treatment.** (**A**) The intracellular ROS production was measured with DCFH-DA after GBC-SD and NOZ cells were treated with or without 1 mM melatonin for 48 h. (**B**) Pre-treatment with 2 mM NAC for 1 hour significantly inhibited the production of ROS in GBC-SD and NOZ cells treated with 1 mM melatonin. (**C**, **D**) Pre-treatment with 2 mM NAC for 1 hour reversed the inhibition effects of melatonin in GBC-SD and NOZ cells. Three biological replicates were performed. Data are presented as mean ± SD. Mel, melatonin; ^****^P < 0.0001; ns, no significance.

To investigate the anti-proliferation mechanisms of melatonin on gallbladder cancer cells, Annexin V and PI double staining apoptosis kit was used for detection of apoptosis in melatonin (1 mM) treated tumor cells for 48 h (Figure 3A). Melatonin significantly increased the early and late apoptotic ratio in NOZ and GBC-SD cells (Figure 3B). Western blot analysis revealed that 1 mM melatonin treatment markedly elevated the expression levels of apoptosis-associated proteins, including Bax, cleaved PARP, Cytochrome C, and cleaved caspase-3 in GBC-SD as well as NOZ cells (Figure 3C–3F). Besides, melatonin decreased the expression of anti-apoptosis related protein Bcl-2 in both GBC-SD cells and NOZ cells. ROS generation in cells was detected using the ROS assay kit. The ROS levels were increased following 1 mM melatonin treatment for 48 h (Figure 4A). After pre-treatment with 2 mM of the ROS scavenger, N-acetyl-L-cysteine (NAC) for 1 hour, the production of ROS was significantly inhibited in gallbladder cancer cells (Figure 4B). Furthermore, pre-treatment with NAC reversed the inhibitory effects of melatonin on GBC-SD (Figure 4C) as well as NOZ cells (Figure 4D). These results demonstrated that melatonin induced apoptosis by increasing ROS production in gallbladder cancer cells.

### Melatonin induces cell death via suppression of the PI3K/Akt/mTOR signaling pathway

**Figure 5:**
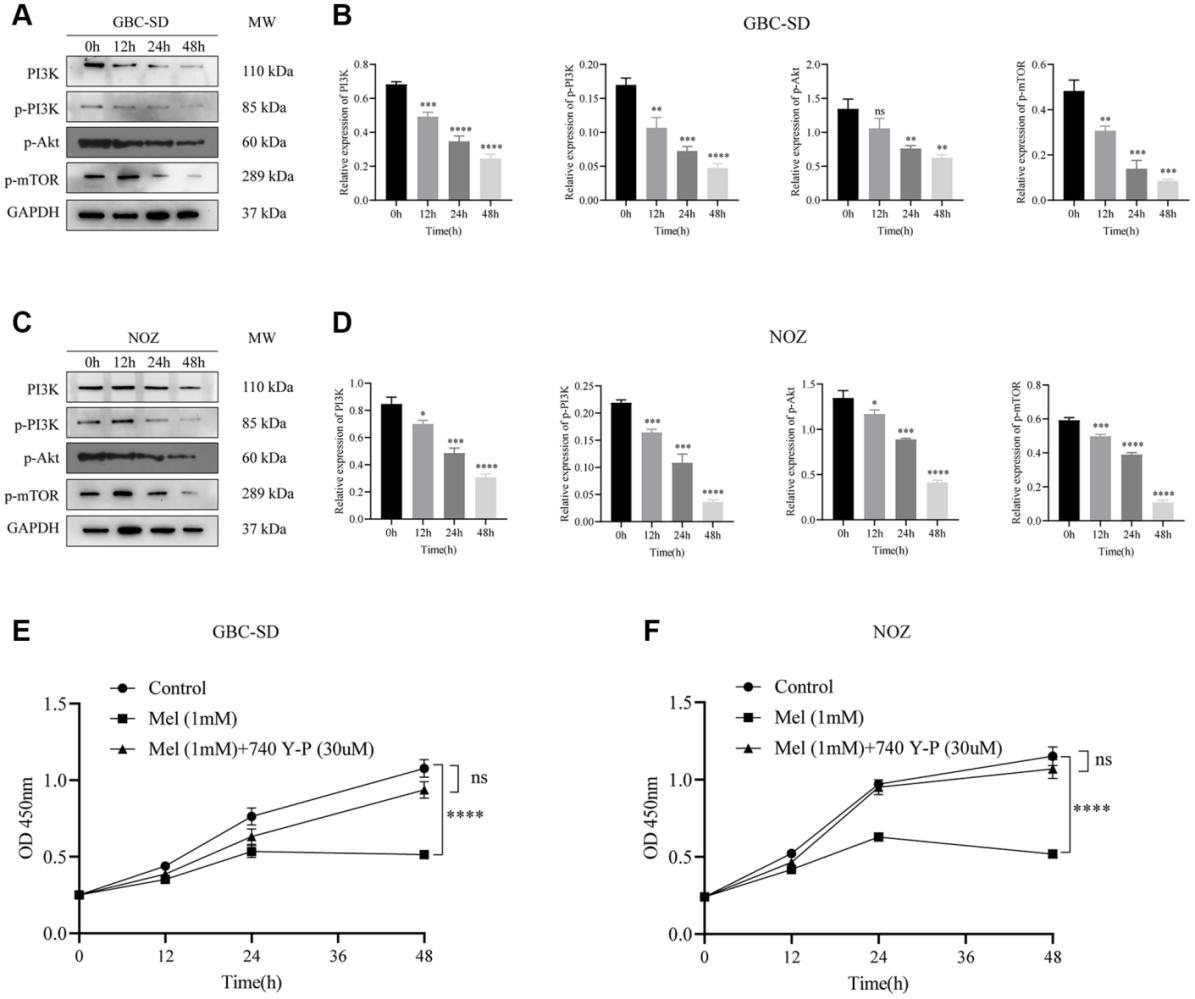
**Melatonin suppresses the activation of the PI3K/Akt/mTOR signaling pathway.** (**A**) The protein expressions of PI3K, p-PI3K, p-Akt, and p-mTOR were detected by Western blotting after GBC-SD cells were treated with 1 mM melatonin for 0, 12, 24, and 48 hours. (**B**) Relative protein expressions were quantified in GBC-SD cells. (**C**) The protein expressions of PI3K, p-PI3K, p-Akt, and p-mTOR were detected by Western blotting after NOZ cells were treated with 1 mM melatonin for 0, 12, 24, and 48 hours. (**D**) Relative protein expressions were quantified in NOZ cells. (**E**, **F**) The inhibitory effects of 1 mM melatonin in GBC-SD and NOZ cells were undermined after co-treatment with a PI3K activator 740 Y-P (30 uM) for 48 h. Three biological replicates were performed. Data are presented as mean ± SD. Mel, melatonin; ^****^P < 0.0001; ^***^P < 0.001; ^**^P < 0.01; ^*^P < 0.05.

The PI3K/Akt/mTOR signaling pathway has been reported to participates in cancer cells proliferation and metastasis. Following 1 mM melatonin treatment at varying times (0, 12, 24, and 48 h), expressions of phosphorylated Akt, PI3K, and mTOR in GBC-SD cells were detected by western blot assay (Figure 5A). Phosphorylation levels of key proteins were time-dependently markedly inhibited (Figure 5B). In the NOZ cells treated with 1 mM melatonin, the phosphorylation levels of Akt, PI3K, and mTOR were also curbed (Figure 5C, 5D). Furthermore, co-treatment with melatonin and a cell-permeable PI3K activator 740 Y-P noticeably undermined the suppressive effects of melatonin on tumor cell proliferation (Figure 5E, 5F). These findings imply that melatonin initiated NOZ and GBC-SD cell apoptosis by suppressing PI3K/Akt/mTOR signaling pathway activation.

### Melatonin inhibits tumor growth *in vivo*

**Figure 6:**
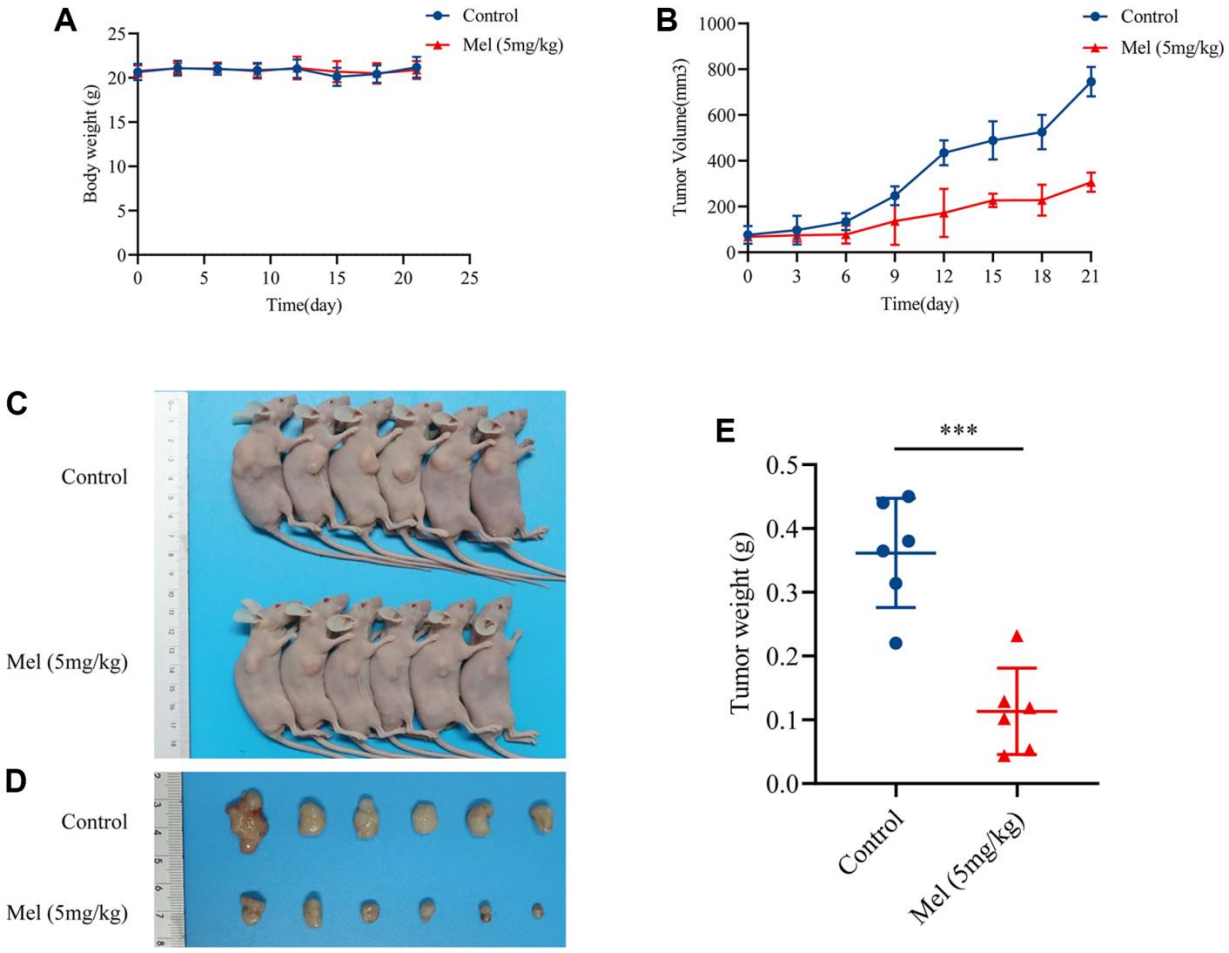
**Melatonin inhibited GBC-SD cells proliferation *in vivo*.** (**A**) Body weights of all mice were recorded every three days (*n* = 6). (**B**) The tumor volume was measured every three days (*n* = 6). (**C**, **D**) The pictures of mice and harvested tumors (*n* = 6). (**E**) Tumor weight measurements (*n* = 6). Data are presented as mean ± SD. Mel, melatonin; ^***^P < 0.001.

Given the potential antitumor effects of melatonin in gallbladder cancer cells, the inhibitory effect *in vivo* was further investigated using an athymic nude mouse model. Interestingly, there was no marked change in body weights over the experimental period (Figure 6A), meaning that melatonin had limited side effects *in vivo*. The mice treated with melatonin (5 mg/kg) showed a significant reduction in tumor volume (Figure 6B) when compared with control groups (Figure 6C, 6D). And the tumor weight eventually decreased after the treatment with 5 mg/kg melatonin (Figure 6E). Taken together, findings imply that melatonin suppressed tumor growth *in vivo* and might provide possible therapeutic options for gallbladder cancer.

## DISCUSSION

Gallbladder cancer has been ranked as the fifth most common gastrointestinal malignancy throughout the world, and it has a very poor prognosis [23, 24]. Most gallbladder cancer patients are diagnosed in advanced stages when the tumor is unresectable owing to the rapid growth and metastasis [25]. Despite the recent advances in medical treatment, several multicenter studies report that the median survival time of GBC is approximately 25 months [26–28]. Novel and effective drugs are urgently required for the treatment of gallbladder cancer. Melatonin is a well-known hormone that is generated by the pineal gland [29]. Recently, the antitumor effects of melatonin have gained significant attention and numerous studies reveal that melatonin exerts growth inhibition on tumor cells [9, 30, 31]. The potential mechanisms include stimulation of apoptosis, regulation of cancer immunity, cell cycle arrest, and modulation of pro-survival signaling [32]. However, limited studies have reported the mechanisms of melatonin action in gallbladder cancer. We confirmed the inhibitory effects of melatonin on GBC-SD and NOZ cells. Further research focusing on the PI3K/Akt/mTOR signaling pathway was also conducted to illustrate the probable mechanism of melatonin.

Apoptosis (Type Ι Programmed Cell Death) is thought to be an important component of various cellular processes that is regulated by extrinsic or intrinsic apoptotic pathways [33, 34]. The Bcl-2 gene family is reported to play a crucial apoptotic role and the Bax/Bcl-2 ratio is an essential indicator [35, 36]. ROS is mainly produced by the mitochondria and scavenged by multiple antioxidants, such as glutathione [37]. Generally, the redox state in the cell is balanced by ROS production and scavenging [38]. When excessive ROS is accumulated, intrinsic pathway of apoptosis is activated while cytochrome C is secreted by the mitochondria to active the downstream of the caspase cascade reaction [39, 40]. It was long believed that melatonin might serve as a potent ROS scavenger. Melatonin was used to protect the pancreatic damage via the decrease of oxidative damage and inflammatory response in the acute pancreatitis [41, 42]. Under certain conditions, however, melatonin is documented to increase ROS production, especially in cultured tumor cells.

For example, treatment with 1 mM melatonin induced intracellular ROS production and apoptotic cell death in tumor leucocytes [43]. And Uguz et al. (2017) showed that melatonin enhances the cytotoxicity of the chemotherapeutic drugs in pancreatic AR42J cells by increasing ROS levels [44]. Also, it was reported that incubation with 1 mM melatonin resulted in decreased cell viability and enhanced ROS production in Hep G2 cells, which was basically consistent with our results [45]. In the present study, melatonin elevated intracellular ROS level, increased apoptotic cell death, and thus suppressed cell viability in gallbladder cancer cells. Cytochrome C, Bax, and cleaved caspase-3 protein levels were time-dependently upregulated after treatment with 1 mM melatonin, while the expression levels of Bcl-2 decreased. Moreover, pre-treatment with NAC for 1h significantly reversed the inhibitory effects of melatonin on NOZ as well as GBC-SD cells. Taken together, our study suggested that melatonin could induce ROS-mediated apoptosis of gallbladder cancer cells. And melatonin might act as a modulator of the cellular redox status, not only a intracellular antioxidant.

Previous studies have shown that various factors and multiple pathways participate in tumorigenesis, including the PI3K/Akt/mTOR signaling pathway [46]. Numerous targeted PI3K suppressors have been evaluated in clinical trials, such as idelalisib for blood cancers [47]. Activation of PI3K mutations has been observed in renal cell cancer, bladder cancer, breast cancer, and so on [48, 49]. Akt and mTOR are the downstream targets of PI3K and abnormal activation often results in the over-proliferation of tumor cells. This study demonstrated that melatonin suppressed PI3K, Akt, as well as mTOR phosphorylation. Moreover, melatonin and 740 Y-P cotreatment weakened the antitumor effects of melatonin. Melatonin suppressed PI3K/Akt/mTOR signaling pathway activation and thus induced NOZ and GBC-SD cells apoptosis.

**Figure 7:**
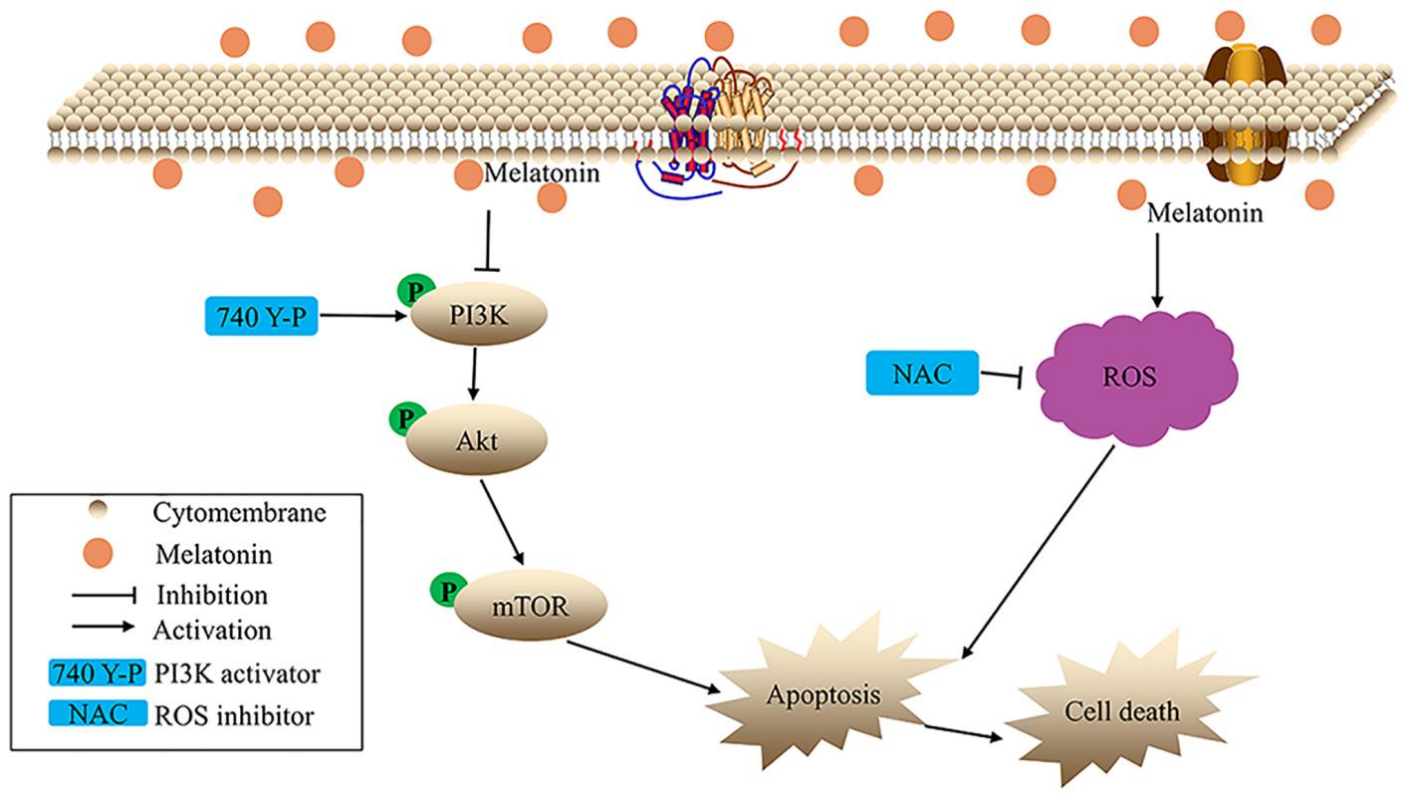
The hypothetical schema of melatonin in gallbladder cancer cells.

Briefly, the present study demonstrates that melatonin suppresses proliferation, migration, as well as invasion of gallbladder cancer cells. Mechanistically, *in vitro*, melatonin promoted ROS-mediated apoptosis of gallbladder cancer cells. Further studies suggest that melatonin suppresses the phosphorylation of the PI3K/Akt/mTOR signaling pathway (Figure 7). Moreover, melatonin also inhibits tumor growth *in vivo* without obvious toxicity. Overall, melatonin may be an effective and novel candidate for the treatment of gallbladder cancer.
